# Supplementary material for: The time-dependent changes in a mouse model of traumatic brain injury with motor dysfunction
Source: PLoS One. 2024 Sep 6;19(9):e0307768. doi: 10.1371/journal.pone.0307768 (PMC11379277; doi:10.1371/journal.pone.0307768)
Supplement: S1 Fig — Related with Fig 4. (A) The Bax and the β-actin of Bax. (B) The Bcl-2 whole membrane and their β-actin. (C) The Mcl-1 and the their β-actin. All data was divided with β-actin and normalized with control group. (DOCX) [file pone.0307768.s001.docx]

**Supplementary information**


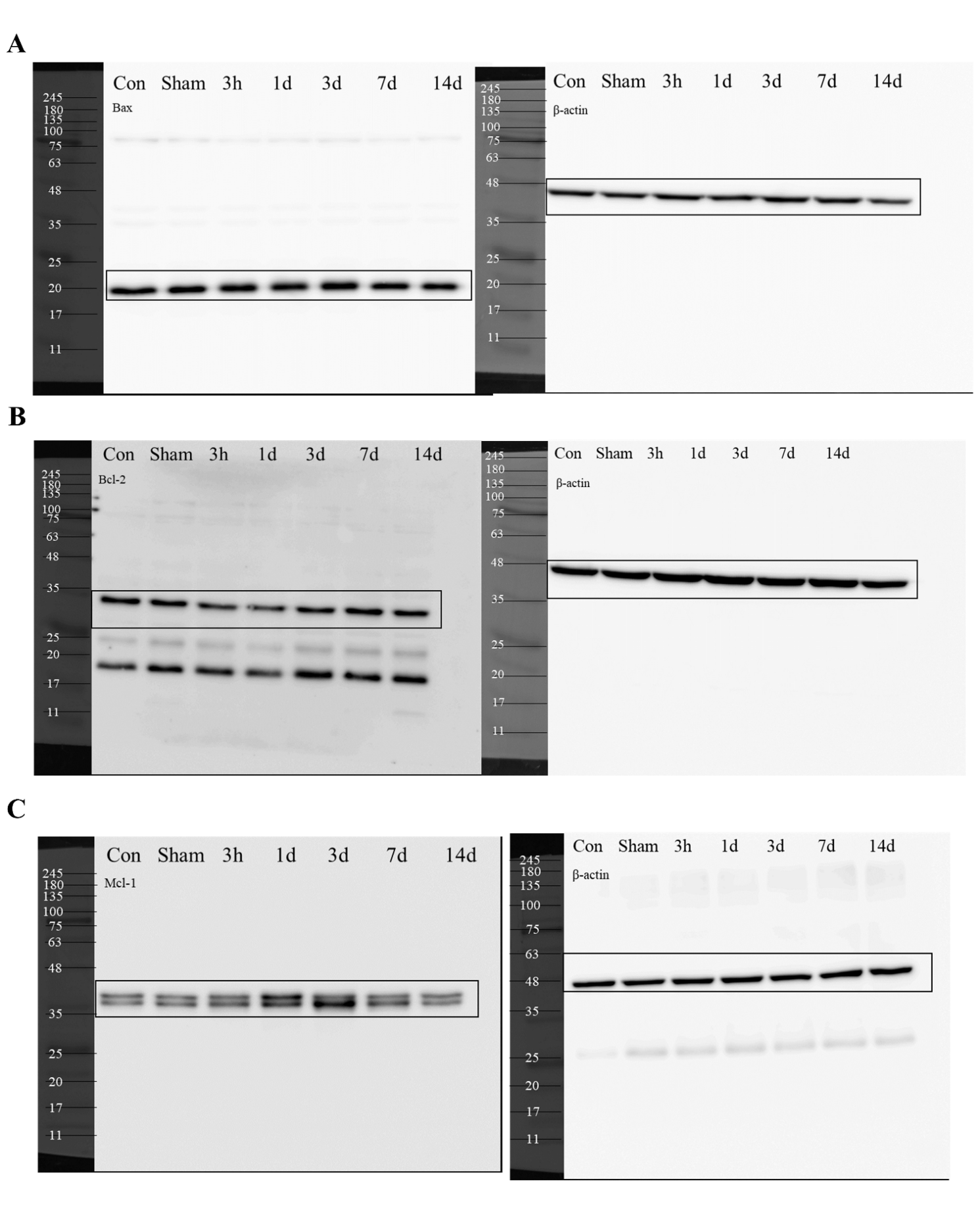


**Supplementary Figure 1**. The whole membrane image of Bcl-2 family. Related with **Figure 4**. **A*,*** The Bax and the β-actin of Bax. **B*,*** The Bcl-2 whole membrane and their β-actin. **C*,*** The Mcl-1 and the their β-actin. All data was divided with β-actin and normalized with control group.
