# Supplementary material for: The time-dependent changes in a mouse model of traumatic brain injury with motor dysfunction
Source: PLoS One. 2024 Sep 6;19(9):e0307768. doi: 10.1371/journal.pone.0307768 (PMC11379277; doi:10.1371/journal.pone.0307768)
Supplement: S2 Fig — Related with Fig 5. (A) The cleaved caspase3, caspase3 and the β-actin of them. (B) The ROCK1 whole membrane and their β-actin. The β-actin was expressed same sample of ROCK1. (C) The p53 and the their β-actin. All data was divided with β-actin and normalized with control group. (DOCX) [file pone.0307768.s002.docx]

**Supplementary information**


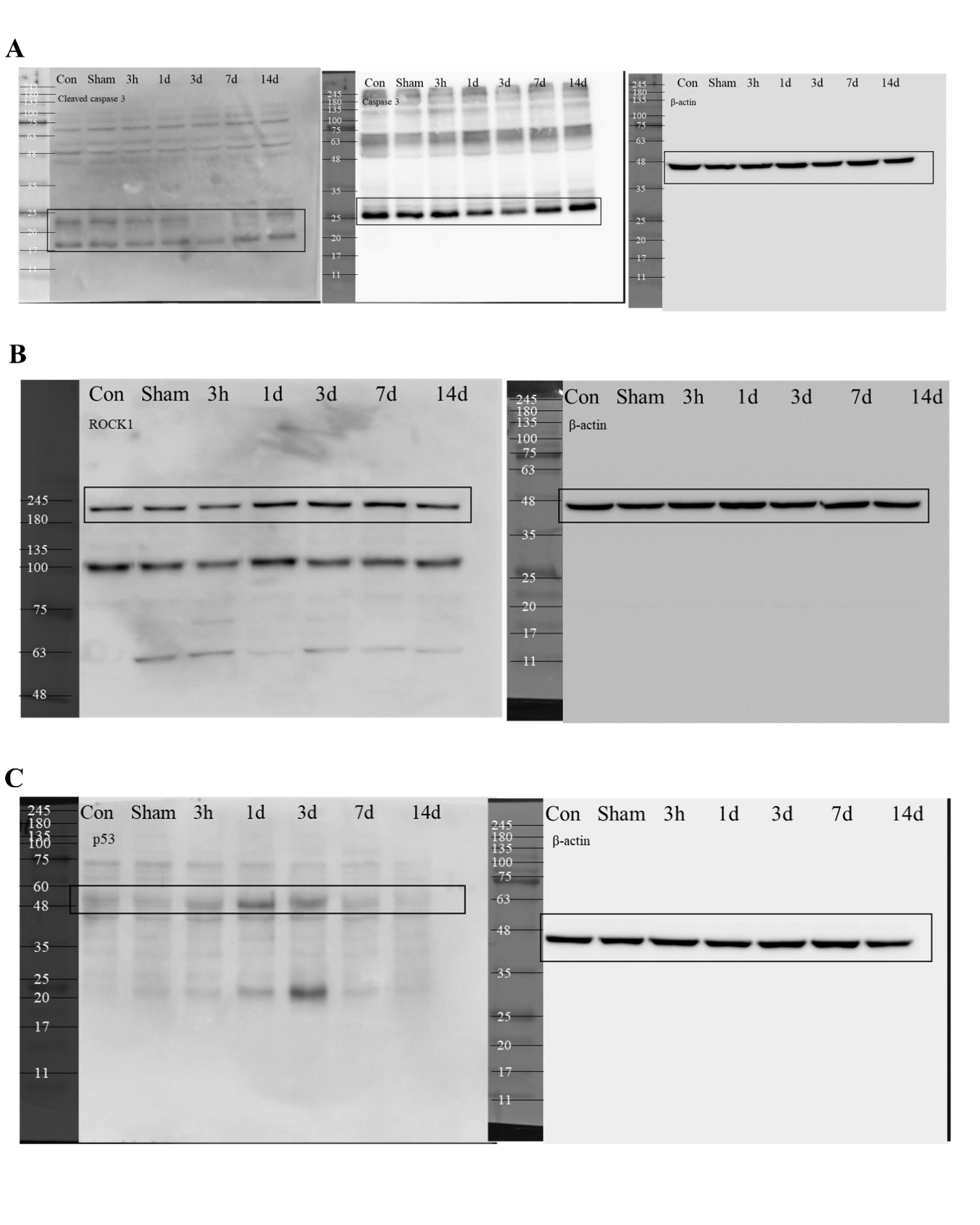


**Supplementary Figure 2.** The whole membrane image of downstream factor of apoptosis signaling. Related with **Figure 5**. **A*,*** The cleaved caspase3, caspase3 and the β-actin of them. **B*,*** The ROCK1 whole membrane and their β-actin. The β-actin was expressed same sample of ROCK1. **C*,*** The p53 and the their β-actin. All data was divided with β-actin and normalized with control group.
