# Supplementary material for: The time-dependent changes in a mouse model of traumatic brain injury with motor dysfunction
Source: PLoS One. 2024 Sep 6;19(9):e0307768. doi: 10.1371/journal.pone.0307768 (PMC11379277; doi:10.1371/journal.pone.0307768)
Supplement: Attachment — Submitted filename: Response to Reviewers.docx [file pone.0307768.s003.docx]

**Comments from Reviewer 1.**

In this manuscript, the authors investigated behavioral and neuropathological changes following a controlled cortical impact (CCI) injury in mice. The study is well documented and does replicate bodies of literature. However, there are many claims should be re-visited. Below are a few major points outlined to be considered.

*I appreciate your detailed review and valuable comments. All the sentences that I changed following your recommendation the instruction of the journal, we have marked as blue color in the manuscript.*

Question 1:

Although claimed as mild TBI injury, there are substantial tissue damage, loss, and behavioral alterations, especially visualized from Fig. 3. Therefore, it should be more suitable to refer to as a TBI model or simply a CCI model.

*Answer: Thank you for your detailed review and valuable comments. Following your recommendation, we have changed TBI model instead of mild TBI model in all the manuscript.*

Question 2:

Some original references regarding the CCI model are missing, such as PMID: 7629863 and 1787745.

*Answer: Thank you for your detailed review and valuable comments. Following your recommendation, we have added the original references in the Introduction and Materials and Methods and ordered all the references.*

Question 3:

One related point is that while it is claimed as a “more concise and effective” mild TBI model, this does not appear to be much more different than numerous established mouse CCI models embedded in literature. Indeed, the behavioral observations along with the histological findings are not new. These may include locomotor dysfunctions, increases in apoptosis, loss of neuronal markers, etc after injury, which are all well characterized in many other studies. This is a major disadvantage of the study that only replicate but not significantly advance understanding of the disease pathophysiology.

*Answer: Thank you for your detailed review and valuable comments. In this study, we evaluated the mouse model which had an accurate area of damage with 2.0 mm of diameter in the brain. Following the results, the mice showed same motor dysfunction with increased apoptotic signaling pathway. In many studies including our results, there are still remain limitations, however, our model showed very concise and delicate sign and symptom than others.*

**Comments from Reviewer 2.**

the manuscript is well drafted and alot of detais is ecountered but there is minimum omission should be deal with them before puplications.

*I appreciate your detailed review and valuable comments. All the sentences that I changed following your recommendation, we have marked as blue color in the manuscript.*

Question 1:

the photo for histopathology and immunohistochemistry should be increasing resolution and increases the description on them.

*Answer: Thank you for your detailed review and valuable comments. Following your recommendation, we have increased the resolution and provided more detail description of the figures.*

Question 2:

the paper is good but need to improve the quality of images

*Answer: Thank you for your detailed review and valuable comments. Following your recommendation, we have increased the quality of images. We will provide improved quality of images, separately.*

Question 3:

western blotting has double bands indicate non specific binding in MCL2

*Answer: Thank you for your detailed review and valuable comments. Our previous studies, the western blotting analysis of MCL2 had showed double bands, so we described and provide the references of the results.*

Question 4:

is there is dimenstion or previous report for stereotaxic injection that you have applied

*Answer: Thank you for your detailed review and valuable comments. In this study, we used the stereotaxic system to calculate the exact region of impact not to inject. For the next work, we are working about the therapeutic effect of drugs in the TBI model to use a stereotaxic, which administered by intracerebroventricular injection with 10 ul of Hamilton syringe (701N, Lot # 697293, Hamilton Company, NV, USA) on a stereotaxic syringe pump (KD Scientific Inc., MA, USA).*

Question 5:

mice genetics should be demostrated and why you shoose this mouse starin .

*Answer: Thank you for your detailed review and valuable comments. Following your recommendation, we have added the reference and the reason to choose the mouse strain in the Materials and Methods.*
